# Determinants of CO_2_ emissions and economic progress: A case from a developing economy

**DOI:** 10.1016/j.heliyon.2022.e12303

**Published:** 2023-01-03

**Authors:** Muhammad Umar, Muhammad Yousaf Raza, Yan Xu

**Affiliations:** a School of Economics and Management, East China Jiaotong University, Nanchang, Jiangxi, 330013, China; b School of Economics, Shandong Technology and Business University, Yantai, Shandong, 255000, China; c School of Mathematics, Ocean University of China, Qingdao, Shan Dong, 266100, China

**Keywords:** Energy consumption, Export, Capital formation, CO_2_ emission, Pakistan

## Abstract

This study attempts to explore the bond between Pakistani exports, gross capital formation, energy use and carbon dioxide emission. It uses the data from Pakistan spanning over a longer time horizon of 40 years ranging from 1981 to 2020. The results of ARDL analysis show that Pakistani exports have inverse relationship with CO_2_ emission in both short as well as long run and the carbon emission reverts to equilibrium at the speed of 54.9%. Increase in carbon emission also lowers export but the causal relationship is only from exports to carbon emission. Energy utilization results in higher carbon emission both in short as well as long run. Based on above findings this study suggests that Pakistan should increase its exports to improve the position of its balance of payment because higher exports do not harm environment in case of Pakistan.

## 1 Introduction

Pakistan is one of the emerging countries in South Asia. It is culturally rich, have ideal weather, and a vast potential in energy sector, a huge portion of which has not been utilized properly [22]. Lessening the carbon dioxide (CO_2_) emissions is imperative due to the low carbon economy is necessary to short-run sustainable growth in the entire world [12]. Thus, the government involvement in lessening CO_2_ emitted is compulsory to attain a low carbon economy [28]. For this, several countries introduced carbon taxes [4] and CO_2_ emissions related trading [10]. Currently, Pakistan has adopted “renewable energy visions-2025 and 2035” that usually target huge carbon emitting sectors (i.e., industrial, agriculture and transport) [23]. These plans comprise a reference to adopt a production way that produces lower carbon content through efficient energy consumption and available resources [23]. No doubt, this would be an expensive investment, which further depends on the ability of various companies to implement the instructions and cover the capital cost.

Pakistan is one of the imperative growing economies, which has substantial economic growth together with India, Bangladesh, and China with global economic development rankings. The impacts of CO_2_ emissions, energy utilization on sustainable economic development are correspondingly grew and raised the pollution in the country. For this, Pakistan has also signed the Paris agreement COP-21with aims to lessen CO_2_ emissions and attaining green economic development [22]. Pakistan has signed energy related projects based on China Pakistan Economic Corridor (CPEC) with China, who also signed for the low carbon and sustainable economic growth goals. Thus, the study analyzes CO_2_ emissions, country's exports, energy consumption, and gross capital formation in Pakistan in a comprehensive way since it is not safe from these issues. The study also answers the driving forces under the growth route of Pakistan's economy by analyzing the association among the stated factors. The research includes gross capital formation, exports, and energy use as independent variables to check the robustness of the model to be employed. The general hypotheses of the study are as follows:

First, huge energy consumption, exports, and lower CO_2_ emissions lead to economic development in Pakistan. Second, with the rise of export, the CO_2_ emissions would decline in the short run. The contribution of the present research is done by the scaling of used variables employing gross capital formation (GCF) and related variables. The export variable is imperative in case of carbon emissions and economic development. We are sure that, in the current study where gross domestic product (GDP), GCF, CO_2_ emission, export, and energy consumption have been widely estimated in the environmental Kuznets curve (EKC) to analyze the presence of the inverted U-shaped relationship in our variables in Pakistan during 1980-2020. For example, numerous studies who investigated the EKC are [3], [5], [8], [9], [45], [46]. Few of the studies established to the presence of the EKC and some did not. Although, some studies investigated GDP per capita in relation to CO_2_ emission, GCF, energy use, imports, and exports simultaneously.

The significance of current study is in its robust empirical analysis and theoretical consideration on the basis of causality relationships on GDP in relation to CO_2_ emission, GCF, exports, and energy utilization in the context of Pakistan over the period of 1980-2020. Realistic investigations have not provided help to reasonable hypothesis on environmental researches, which is directly linked to our estimated variables. Therefore, to the best of our understanding, this is a novel contribution to literature related to the EKC.

The main objectives of the current study are: the study employs the autoregressive distributive lag (ARDL) model so as to run the short and long-run associations among the variables, which are in line with the [33] based on assumptions as well. This model is imperative and a single equation model that performs well for small samples. There are several advantages of the ARDL model over the other cointegration methods: (i) It has well-finite sample properties. (ii) It is different from the Engle and Granger's two-step and Johansen's maximum likelihood methods that sustain from small sample bias. (iii) This technique is relatively flexible on the I(0) or I(1) series and a group of the ‘2’, and preliminary tests for the order of integration of variables are generally not a precondition. Therefore, this method is the best fit for the current measurement based on a sample data.

We use the ARDL method in the following ways: first, we investigate for cointegration to calculate that there is some long-run relationship among the factors. If we found the cointegration then we estimate both short and long-run relationships. This is because, an error correction model can be seen from the ARDL method using a simple linear transformation, this method is appropriate to investigate the short and long-run factors of methods simultaneously. Thus, these measures are reliably necessary for policymakers and researchers to concentrate and plan the growing energy demand for the country's development. We also conduct a comparison analysis on the carbon emissions with various factors. The current research tried to support the related literature from energy and economic perspective.

As Pakistan is an emerging country, country meets its increasing energy demand and production, this research has tried to concentrate on the energy, economy, and environment on modeling the demand form exports and related factors in Pakistan using the ARDL method. For example, concentrating on the specific region of Pakistan, we systematically analyze the nexus between CO_2_ emissions and related factors, which will help policymakers, express specific and effective trade, economic and energy related policies to lessen CO_2_ emission and obtain sustainable green growth. Moreover, we have analyzed the impact of CO_2_ emission-export relationship for country's environmental situation, which will help traders, government and businesses. This will give new insights for scholars and policymakers who focus on the nexus between international trade and carbon emissions in trading countries.

Finally, study provides the short run and long run and causality relationships among the variables which will further help policymakers. The basic objectives of present study are to model an empirical discussion about the relationship between CO_2_ emission, export of goods and services, gross capital formation and energy consumption for the period of 40 years. The causality relationship will further discuss the major role of export to carbon emission. Based on literature and best of our knowledge, no empirical study seems to have analyzed the demand for existing factors for Pakistan's current period. In addition to the research questions and primary contribution to the literature are as follows: what are the impacts of energy consumption that actually lead to economic development in Pakistan? What are the impacts of Pakistan's exports in lowering CO_2_ emissions in short and long run analysis? What are causal relationships between various factors during the study period? What are the impacts of higher exports on Pakistan's balance of payment and environment? What are the most important policies to distinguish to check renewable energy consumption in Pakistan?

The remaining part of the study is as follows: section 2 provides a literature review, section 3 provides data and methods and section 4 provides empirical results and discussion. The conclusion and policy recommendations are provided in section 5.

## 2 Literature review

The association between GDP, energy consumption, export, GCF, and CO_2_ emission has become a topic of discussion over the past decade [1]. Numerous studies have described that, notwithstanding the advantageous impact of these variables, and they also have negative results on the environment. Although, this is very acute to the extent number of scholars and policymakers have dedicated much time to finding a final solution to it [11], [16].

The literature includes the four main strands in the current study. First, the nexus between energy consumption (EC) and economic growth (EG) is huge and has been abundantly studied under ‘4’ testable hypothesis, i.e., EC-EG (known as growth hypothesis), EG-EC (known as conservation hypothesis), bidirectional association between EG and EC (known as feedback hypothesis), and no relationship (known as neutrality hypothesis) [18], [32]. Second, the literature on the association between EG and CO_2_ emission is also well-established by analyzing the presence of the EKC hypothesis [2], [17], [21], [42]. The EKC hypothesis considers an inverted U-shaped relationship between EG and carbon emissions. Third, the literature combines the previous strands by analyzing dynamic association between CO_2_ emissions, EC and EG in the trivariate structure, for example, [6], [48], [47] found the mixed results. Moreover, the mixed and variations in outcomes are common in these researches because of various econometric techniques, regions, country, institutions, variables, and time period selections [37], [40], [41]. Finally, the literature extends the third strand by integrating extra variables and is a developing area of research. Our research belongs to this kind where we analyze the impacts of GCF, GDP, export, and EC on CO_2_ emission in Pakistan employing the ARDL framework. An export and GCF as an added variable, especially for Pakistan that GDP and EC show significant relationship with CO_2_ emissions, for example, [15] assessed that imports add to raised CO_2_ emissions while exports significantly lessen CO_2_ emissions in a country. Liu and Bae [25] investigated that a one percent increase in real GDP raises CO_2_ emissions by 0.6%, by long-run feedback granger causality among industry, CO_2_ emissions, and GDP in China. Chen et al. [13] analyzed that there is a bidirectional causality between EC, CO_2_ emissions and EG in the long-run for the northern, southern and western regions in each country panel analysis. Shahbaz et al. [39] analyzed the association between EC and EG, including capital and trade for the production possibility frontier of India, China, Russia, Brazil, and African countries, employing the cointegration analysis. They found the presence of long-run equilibrium relationship among the variables. Moreover, the GCF was estimated to rise EG and trade openness encouraged growth. Overall, few feedback signs were found between biomass EC and EG. Further, it is evident from the studies of Yang and Zhao [50] for India and estimated that trade openness and CO_2_ emissions have a direct relationship; Shahbaz et al. [38] found that EG and EC raised CO_2_ emissions, while EG and trade openness lessened CO_2_ emissions in Indonesia. Similarly, [34], [35] found bidirectional causality between EG and CO_2_ emissions in Bangladesh, Indonesia and Pakistan.

From the perspective of Asian economies, Muhammad and Khan [30] investigated that EC, FDI, CO_2_ emissions, and capital played an imperative role in the EG of Asian countries. Nasreen et al. [31] investigated 18 Asian countries and found that CO_2_ emissions rose due to transport EC and EG, while Rahman [34] analyzed the similar outcomes that EC and exports increased the CO_2_ emissions in case of 11 Asian populous nations. Akalpler and Hove [2] was the only study that explored the EC, EG, imports, exports, and environmental issues in India for the period of 1971-2014. The objective was to find the causal relationship between variables. Thus, it is obvious from the literature provided above that our study makes unique and new contribution to the present literature, including latest period of 1980-2020, which is ignored by current studies. The study provides the literature on the nexus of EC, EG, GCF, exports, and CO_2_ emissions for Pakistan. Also, being the first and latest study focused on latest sample, framework, hypothesized within EKC framework measures the country's current situation.

Finally, in the current state of art, we find that the studies in Pakistan are very limited. The empirical literature is quiet about the evaluation of the spatial effects of macroeconomic variables on the CO_2_ emissions in Pakistan. In the review on the EKC hypothesis, this dependency is limited for Pakistan as well as limited in the other regions in the world, therefore, current study tried to fill this gap by analyzing the EKC hypothesis in this emerging region in the context of major variables stated above.

## 3 Data and methodology

### 3.1 Data

The data regarding all the variables was obtained from the World Development Indicators of The World Bank. The data regarding all the variables range from 1981 to 2020, a period of 40 years. Carbon dioxide emission metric tons per capita (CO2EM_MTPC) represent the CO_2_ emission from the burning of fossil fuel. It includes the CO_2_ produced during consumption of solid, liquid, and gas fuels and gas flaring. Exports of goods and services (EXPGS_GDP) represent the value of all goods and other market services provided to the rest of the world. They include the value of merchandise, freight, insurance, transport, travel, royalties, license fees, and other services, such as communication, construction, financial, information, business, personal, and government services. They exclude compensation of employees and investment income (formerly called factor services) and transfer payments.

Gross capital formation to GDP ratio (GCF_GDP) shows a random walk with drift. The series shows crests and troughs and shows a declining trend overall. Energy use kilogram of oil equivalent per capita (ENGU_PC) refers to use of primary energy before transformation to other end-use fuels, which is equal to indigenous production plus imports and stock changes, minus exports and fuels supplied to ships and aircraft engaged in international transport. The descriptive statistics regarding all the variables are given in Table 1 given in the results section.

### 3.2 Methodology

To explore the relationship between different economic factors and CO_2_ emission in Pakistan, number of steps were followed. In the first step, graphs of all the series were drawn to see whether they show a trend or a random walk with drift. Based on observed behavior, augmented Dickey Fuller tests (ADF), Phillips-Perron test (PP) and Kwiatkowski Schmidt Shin test (KSS) were run to know whether the series were stationary or not. Augmented Dickey Fuller test has the null hypothesis stating that there exists a unit root. Rejection of null hypothesis in Dickey Fuller test means that the series does not have unit root i.e., it is stationary. On the other hand, failure to reject null hypothesis means that series has a unit root i.e., it is not stationary and have a trend component. The results of ADF stationarity test of all the series are given in Table 3. As per the results of ADF test, exports to GDP ratio and gross capital formation as a percentage of GDP are stationary at level but CO_2_ emission and energy use per capita become stationary at first difference.

To be prudent, we have also run Phillips-Perron test (PP) and Kwiatkowski Schmidt Shin test (KSS) to check for the stationarity of different series. As per the results of PP test, none of the series is stationary at level and all the series are stationary at first difference. The difference between ADF and PP tests is that, as per the results of ADF, exports to GDP ratio and gross capital formation to GDP ratio are stationary at level but PP test results show that they become stationary at first difference. The findings of KSS test lie between ADF and KSS. As per KSS test, all the series are stationary at first difference except GCF_GDP which is stationary at level. As the dependent variable is stationary at first difference and the independent variables are either stationary at level or first difference, so the ARDL seems to be an appropriate technique for the analysis. Therefore, this study explores the short as well as long-run relationship between variables using ARDL technique.

ARDL is a model based on Ordinary Least Square (OLS) mechanism and it can be applied to both stationary as well as non-stationary time series and even if the series do not have same level of integration. In an ARDL model, dependent variable is regressed on its own lagged values as well as the lagged values of all the other variables which are part of the system. In this technique number of equations is equal to the number of variables in a system as every variable is taken as dependent variable and regressed on its own lagged values and lagged values of other variables. The appropriate number of lags is required to capture the data generating process leading from general to specific structure. A dynamic error correction model (ECM) can also be obtained by linearly transforming ARDL. The general form of ARDL (p, q) model is given below in equation (1).

(1) $$Y_{t}=\gamma_{0i}+\sum_{i=1}^{p}\delta_{i}Y_{t-i}+\sum_{i=0}^{q}\beta_{i}^{\prime }X_{t-1}+\varepsilon_{it}$$

Where $Y_{t}$ is the dependent variable and *γ* is the y-intercept. $\delta_{i}$ is the coefficient of lagged values of dependent variable and $\beta_{i}^{\prime }$ are the coefficients of different lagged values of regressors. *i* = 1… k; p, q are optimal lag orders and $\varepsilon_{it}$ is the vector of error terms. The first step in the application of ARDL model is to know whether the series are co-integrated or not. The following equations (2)-(5) are for bounds test of co-integration, the conditional ARDL (p, q_1_, q_2_, q_3_, q_4_, q_5_) with four variables is specified where b_11_, b_21_, b_31_ and b_41_ are coefficients to check for co-integration between the variables. Rejection of these betas means that variables are not co-integrated and vice versa. If the series are not co-integrated, we only specify short run model and long run ARDL model is only applied if the series are found to be co-integrated. The mathematical equations for the bounds test are given below.

(2) $$\Delta CO2EM\_MTPC_{t}=\alpha_{01}+b_{11}CO2EM\_MTPC_{t-1}+b_{21}\mathrm{EXPGS}\_GDP_{t-i}+b_{31}\mathrm{GCF}\_GDP_{t-i}+b_{41}\mathrm{ENGU}\_PC_{t-i}+\sum_{i=1}^{p}a_{1i}\Delta CO2EM\_MTPC_{t-i}+\sum_{i=1}^{q}a_{2i}\Delta EXPGS\_GDP_{t-i}+\sum_{i=1}^{q}a_{3i}\Delta \mathrm{GCF}\_GDP_{t-i}+\sum_{i=1}^{q}a_{4i}\Delta ENGU\_PC_{t-i}+e_{1t}$$

(3) $$\Delta EXPGS\_GDP_{t}=\alpha_{02}+b_{21}CO2EM\_MTPC_{t-1}+b_{22}\mathrm{EXPGS}\_GDP_{t-i}+b_{32}\mathrm{GCF}\_GDP_{t-i}+b_{42}\mathrm{ENGU}\_PC_{t-i}+\sum_{i=1}^{p}a_{1i}\Delta CO2EM\_MTPC_{t-i}+\sum_{i=1}^{q}a_{2i}\Delta EXPGS\_GDP_{t-i}+\sum_{i=1}^{q}a_{3i}\Delta \mathrm{GCF}\_GDP_{t-i}+\sum_{i=1}^{q}a_{4i}\Delta ENGU\_PC_{t-i}+e_{2t}$$

(4) $$\Delta GCF\_GDP_{t}=\alpha_{03}+b_{13}CO2EM\_MTPC_{t-1}+b_{23}\mathrm{EXPGS}\_GDP_{t-i}+b_{33}\mathrm{GCF}\_GDP_{t-i}+b_{43}\mathrm{ENGU}\_PC_{t-i}+\sum_{i=1}^{p}a_{1i}\Delta CO2EM\_MTPC_{t-i}+\sum_{i=1}^{q}a_{2i}\Delta EXPGS\_GDP_{t-i}+\sum_{i=1}^{q}a_{3i}\Delta \mathrm{GCF}\_GDP_{t-i}+\sum_{i=1}^{q}a_{4i}\Delta ENGU\_PC_{t-i}+e_{3t}$$

(5) $$\Delta ENGU\_PC_{t}=\alpha_{04}+b_{14}CO2EM\_MTPC_{t-1}+b_{24}\mathrm{EXPGS}\_GDP_{t-i}+b_{34}\mathrm{GCF}\_GDP_{t-i}+b_{44}\mathrm{ENGU}\_PC_{t-i}+\sum_{i=1}^{p}a_{1i}\Delta CO2EM\_MTPC_{t-i}+\sum_{i=1}^{q}a_{2i}\Delta EXPGS\_GDP_{t-i}+\sum_{i=1}^{q}a_{3i}\Delta \mathrm{GCF}\_GDP_{t-i}+\sum_{i=1}^{q}a_{4i}\Delta ENGU\_PC_{t-i}+e_{4t}$$

Where Δ is the difference operator, t represents time and t-1 represents lagged value of a certain variable. The symbols of variables have already been explained in the data section and e_it_ stands for error term. As per the results, we reject null hypothesis of co-integration for some of the models but not for the others. So, we have run ARDL for short as well as long run. The mathematical models for short run ARDL are given below in equations (6)-(9).

(6) $$\Delta CO2EM\_MTPC_{t}=\alpha_{01}+\sum_{i=1}^{p}a_{1i}\Delta CO2EM\_MTPC_{t-i}+\sum_{i=1}^{q}a_{2i}\Delta EXPGS\_GDP_{t-i}+\sum_{i=1}^{q}a_{3i}\Delta \mathrm{GCF}\_GDP_{t-i}+\sum_{i=1}^{q}a_{4i}\Delta ENGU\_PC_{t-i}+e_{t}$$

(7) $$\Delta EXPGS\_GDP_{t}=\alpha_{01}+\sum_{i=1}^{p}a_{1i}\Delta CO2EM\_MTPC_{t-i}+\sum_{i=1}^{q}a_{2i}\Delta EXPGS\_GDP_{t-i}+\sum_{i=1}^{q}a_{3i}\Delta \mathrm{GCF}\_GDP_{t-i}+\sum_{i=1}^{q}a_{4i}\Delta ENGU\_PC_{t-i}+e_{t}$$

(8) $$\Delta GCF\_GDP_{t}=\alpha_{01}+\sum_{i=1}^{p}a_{1i}\Delta CO2EM\_MTPC_{t-i}+\sum_{i=1}^{q}a_{2i}\Delta EXPGS\_GDP_{t-i}+\sum_{i=1}^{q}a_{3i}\Delta \mathrm{GCF}\_GDP_{t-i}+\sum_{i=1}^{q}a_{4i}\Delta ENGU\_PC_{t-i}+e_{t}$$

(9) $$\Delta GCF\_GDP_{t}=\alpha_{01}+\sum_{i=1}^{p}a_{1i}\Delta CO2EM\_MTPC_{t-i}+\sum_{i=1}^{q}a_{2i}\Delta EXPGS\_GDP_{t-i}+\sum_{i=1}^{q}a_{3i}\Delta \mathrm{GCF}\_GDP_{t-i}+\sum_{i=1}^{q}a_{4i}\Delta ENGU\_PC_{t-i}+e_{t}$$

Where a_1i_, a_2i_, a_3i_ and a_4i_ are short run coefficients and the rest of the symbols have already been explained. The long run ARDL model is given below in equations (10)-(13).

(10) $$\Delta CO2EM\_MTPC_{t}=\alpha_{01}+\sum_{i=1}^{p}a_{1i}\Delta CO2EM\_MTPC_{t-i}+\sum_{i=1}^{q}a_{2i}\Delta EXPGS\_GDP_{t-i}+\sum_{i=1}^{q}a_{3i}\Delta \mathrm{GCF}\_GDP_{t-i}+\sum_{i=1}^{q}a_{4i}\Delta ENGU\_PC_{t-i}+\lambda ECT_{1-1}+e_{t}$$

(11) $$\Delta EXPGS\_GDP_{t}=\alpha_{01}+\sum_{i=1}^{p}a_{1i}\Delta CO2EM\_MTPC_{t-i}+\sum_{i=1}^{q}a_{2i}\Delta EXPGS\_GDP_{t-i}+\sum_{i=1}^{q}a_{3i}\Delta \mathrm{GCF}\_GDP_{t-i}+\sum_{i=1}^{q}a_{4i}\Delta ENGU\_PC_{t-i}+\lambda ECT_{1-1}+e_{t}$$

(12) $$\Delta GCF\_GDP_{t}=\alpha_{01}+\sum_{i=1}^{p}a_{1i}\Delta CO2EM\_MTPC_{t-i}+\sum_{i=1}^{q}a_{2i}\Delta EXPGS\_GDP_{t-i}+\sum_{i=1}^{q}a_{3i}\Delta \mathrm{GCF}\_GDP_{t-i}+\sum_{i=1}^{q}a_{4i}\Delta ENGU\_PC_{t-i}+\lambda ECT_{1-1}+e_{t}$$

(13) $$\Delta ENGU\_PC_{t}=\alpha_{01}+\sum_{i=1}^{p}a_{1i}\Delta CO2EM\_MTPC_{t-i}+\sum_{i=1}^{q}a_{2i}\Delta EXPGS\_GDP_{t-i}+\sum_{i=1}^{q}a_{3i}\Delta \mathrm{GCF}\_GDP_{t-i}+\sum_{i=1}^{q}a_{4i}\Delta ENGU\_PC_{t-i}+\lambda ECT_{1-1}+e_{t}$$

Where $\lambda =(1-\sum_{i=1}^{p}\delta_{i})$ is speed of adjustment with a negative sign and ECT stands for error correction term. ECT captures the long run relationship in the model. *λ* Should be negative and significant to show that the system reverts to long-run equilibrium and if *λ* has a positive sign it means that model is explosive, and it does not converge to the long run equilibrium.

## 4 Empirical findings

Table 1 gives the descriptive statistics for all the variables. The average CO_2_ emission in metric tons per capita is 0.685 with a standard deviation of 0.14. The highest recorded CO2 emission in metric tons is 0.982 and the lower recorded value was 0.426. The annual average of total exports of goods and services to GDP ratio was 13.29% with a standard deviation of 2.377. This ratio has the highest value of 17.56% with a lowest record of 8.25%. Gross capital formation as a percentage of GDP averaged 17.23% with a standard deviation of 1.67. The average value of energy use per capita is 425.69 with a standard deviation of 49.13. The minimum and maximum values for this variable are 328.93 and 500.43 respectively. Table 2 provides the results for pairwise correlation coefficients along with p values in parentheses.

**Table 1:** Description of variables.

	CO2EM_MTPC	EXPGS_GDP	GCF_GDP	ENGU_PC
Mean	0.685	13.292	17.557	425.691
Median	0.684	13.426	17.821	440.800
Std.	0.140	2.377	1.673	49.138
Minimum	0.426	8.257	14.121	328.936
Maximum	0.982	17.271	20.685	500.432
Skewness	0.041	-0.208	-0.321	-0.579
Kurtosis	2.338	2.306	2.021	2.142
N	38	40	40	34

**Table 2:** Pairwise correlation coefficients.

	CO2EM_MTPC	EXPGS_GDP	GCF_GDP	ENGU_PC
CO2EM_MTPC	1			
EXPGS_GDP	-0.295*	1		
	(0.073)			
GCF_GDP	-0.525***	0.395**	1	
	(0.001)	(0.012)		
ENGU_PC	0.980***	0.266	-0.440***	1
	(0.000)	(0.129)	(0.009)	

**Notre**: p-values are in parentheses.

Table 3 provides the results of the augmented Dickey Fuller test used to know about the stationarity of the series [14]. As per the results, CO2EM_MTPC was not stationary at level; however, it became stationary at first difference. On the other hand, total exports to GDP ratio and gross capital formation to GDP ratio are stationary at level. Like CO_2_ emission, energy consumption was not stationary at level, and it became stationary at first difference. As per the results of PP test, none of the series is stationary at level and all the series are stationary at first difference. The difference between ADF and PP tests is that, as per the results of ADF, exports to GDP ratio and gross capital formation to GDP ratio are stationary at level but PP test results show that they become stationary at first difference. The findings of KSS test lie between ADF and KSS. As per KSS test, all the series are stationary at first difference except GCF_GDP which is stationary at level. As all the series are stationary at level or first difference, so we may apply the Autoregressive Distributive Lag model (ARDL) approach for the analysis as none of the series is stationary at the second difference.

**Table 3:** Tests for stationarity of series.

Variable	Level	First difference
**Augmented Dickey Fuller test (ADF)**		
CO2EM_MTPC	-2.681	-6.253***
	(0.244)	(0.000)
EXPGS_GDP	-2.665***	
	(0.005)	
GCF_GDP	-2.145**	
	(0.019)	
ENGU_PC	0.084	-5.541***
	(0.995)	(0.000)
**Phillips-Perron test (PP)**		
CO2EM_MTPC	-0.389	-4.847***
	(0.912)	(0.000)
EXPGS_GDP	-1.348	-6.446***
	(0.607)	(0.000)
GCF_GDP	-1.813	-6.557***
	(0.374)	(0.000)
ENGU_PC	-2.289	-4.508***
	(0.176)	(0.000)
**Kwiatkowski Schmidt Shin test (KSS)**		
CO2EM_MTPC	0.148	0.0985**
	(0.146)	(0.146)
EXPGS_GDP	0.68	0.0445**
	(0.146)	(0.146)
GCF_GDP	0.137**	
	(0.146)	
ENGU_PC	0.592	0.065**
	(0.146)	(0.146)

Test statistics are given for ADF and PP test where the p-values are given in brackets. For KSS test values for zero lag are provided with 5% critical values in brackets.

Table 4 presents the coefficients for short term relationships measured based on the ARDL technique. The CO_2_ emission in metric tons per capita (CO2EM_MTPC) is the dependent variable in model one and exports to GDP ratio is the main independent variable. As per the results, growth in exports adversely impacts carbon emission. An increase in exports is associated with lesser carbon emission. One percent increase in current period's exports to GDP ratio results in lowering CO_2_ emission by 0.008 metric tons per capita. However, previous period's exports do not explain variation in carbon emission at 5% level of significance. Nevertheless, previous period's exports do not affect current period's carbon emission. The current period's gross capital formation does not significantly affect current carbon emission however previous period's GCF results in higher CO_2_ emission. The relationship between energy use per capita and CO_2_ emission is interesting. Increase in current period's energy use results in higher carbon emission but previous year's energy use adversely impacts current carbon emission.

**Table 4:** Short term relationship.

	CO2EM_MTPC (1)	EXPGS_GDP (2)	GCF_GDP (3)	ENGU_PC (4)
CO2EM_MTPC				
L0		-38.5851***	-20.640	254.228***
		(0.002)	(0.112)	(0.000)
L1	0.451**	8.264	5.066	-117.338**
	(0.012)	(0.522)	(0.688)	(0.039)
EXPGS_GDP				
L0	-0.008***	0.474***	-0.293	2.410***
	(0.002)	(0.006)	(0.128)	(0.005)
L1	0.002		0.167	-0.115
	(0.565)		(0.355)	(0.893)
GCF_GDP				
L0	-0.005	-0.308		1.575*
	(0.112)	(0.128)		(0.088)
L1	0.007**	0.521**	0.834***	-2.197**
	(0.028)	(0.015)	(0.000)	(0.027)
ENGU_PC				
L0	0.003***	0.114***	0.071*	
	(0.000)	(0.005)	(0.088)	
L1	-0.001**	-0.035	-0.036	0.615***
	(0.036)	(0.390)	(0.362)	(0.000)
CONS	-0.194	-10.506**	-0.235	54.311**
	(0.012)	(0.049)	(0.965)	(0.025)
N	33	33	33	33
Adj. R-sq	0.983	0.714	0.645	0.9901

Exports to GDP ratio is the dependent variable in model 2. As per the results of short run relationship, current period's carbon emission has significant negative impact on Pakistani exports. However, previous period's emission does not affect exports. GCF_GDP lagged values significantly positively affect current exports but current values of GCF do not explain variation in exports. Current period's energy consumption positively affects exports however previous period's energy use does not explain variation in exports.

Gross capital formation is the dependent variable in model 3. None of the independent variables explain variation in gross capital formation. The results for model 4, where energy consumption is a dependent variable tell a completely different story because all the independent variables are significant determinants of energy use. Current period's carbon emission values significantly positively affect energy use, but previous period's carbon emission values negatively affect energy use. Current values of exports to GDP ratio also positively affect energy use. Lagged values of gross capital formation are also a significant determinant of energy use and GCF_GDP has significant negative relationship with energy use.

Table 5 shows the ARDL and bound test results for long-term relationship between the series. The upper portion of the table presents the results for speed of adjustments which mean that how quickly the entire system reverts back to its long run equilibrium. The lower portion of table offers the long-run coefficients for independent variables. The bottom portion shows F-statistics for bound test and critical values at a 5% level of significance. As per the results of bound test, we reject the null hypothesis of no level relation which implies that there occurs a possible long-run relationship between the variables of model 5. The results for the speed of adjustment obtained in model 5 suggest that the whole system reverts back to long-run equilibrium at a speed of 55%. There exists a long run relationship between exports and carbon emission. Higher exports lead to lower carbonization and vice versa. The relationship between energy use and carbon emission is direct i.e., higher energy consumption leads to more carbon in air in the long run.

**Table 5:** Long term relationship.

	D.CO2EM_MTPC (5)	D.EXPGS_GDP (6)	D.GCF_GDP (7)	D.ENGU_PC (8)
**Adjustment**				
CO2EM_MTPC.L1	-0.549***			
	(0.003)			
EXPGS_GDP.L1		-0.526***		
		(0.003)		
GCF_GDP.L1			-0.166	
			(0.256)	
ENGU_PC.L1				-0.385***
				(0.010)
**Long run relationship**				
CO2EM_MTPC		-57.601***	-93.821	355.584***
		(0.002)	(0.459)	(0.000)
EXPGS_GDP	-0.012***		-0.763	5.960***
	(0.001)		(0.619)	(0.001)
GCF_GDP	0.004	0.404		-1.616
	(0.315)	(0.138)		(0.363)
ENGU_PC	0.003***	0.151***	0.213	
	(0.000)	(0.001)	(0.499)	
CONS	-0.194	-10.506**	-0.235	54.311**
	(0.012)	(0.049)	(0.965)	(0.025)
N	33	33	33	33
Adj. R-sq	0.699	0.326	0.061	0.705
F-statistics	2.959	3.01	0.978	3.201
Critical values at 5%	(I0) I(1)	(I0) I(1)	(I0) I(1)	(I0) I(1)
	3.23 4.35	3.23 4.35	3.23 4.35	3.23 4.35

Bound test results for model 6 also show that there exists a long run relationship among carbon emission, exports and other series. As per the results, exports also get back to long run equilibrium at a speed of 52.6% and carbon emission doe negatively impact exports in long run. The association between energy use and exports is positive in long run. As per the results of model seven, none of the variables explain variation in gross capital formation to GDP ratio. The result of model eight shows that there exists long run relationship between all the series and energy use reverts back to its equilibrium position at a speed of 38.5%. CO_2_ emission and exports have strong positive association with energy utilization.

**Figure 1:**
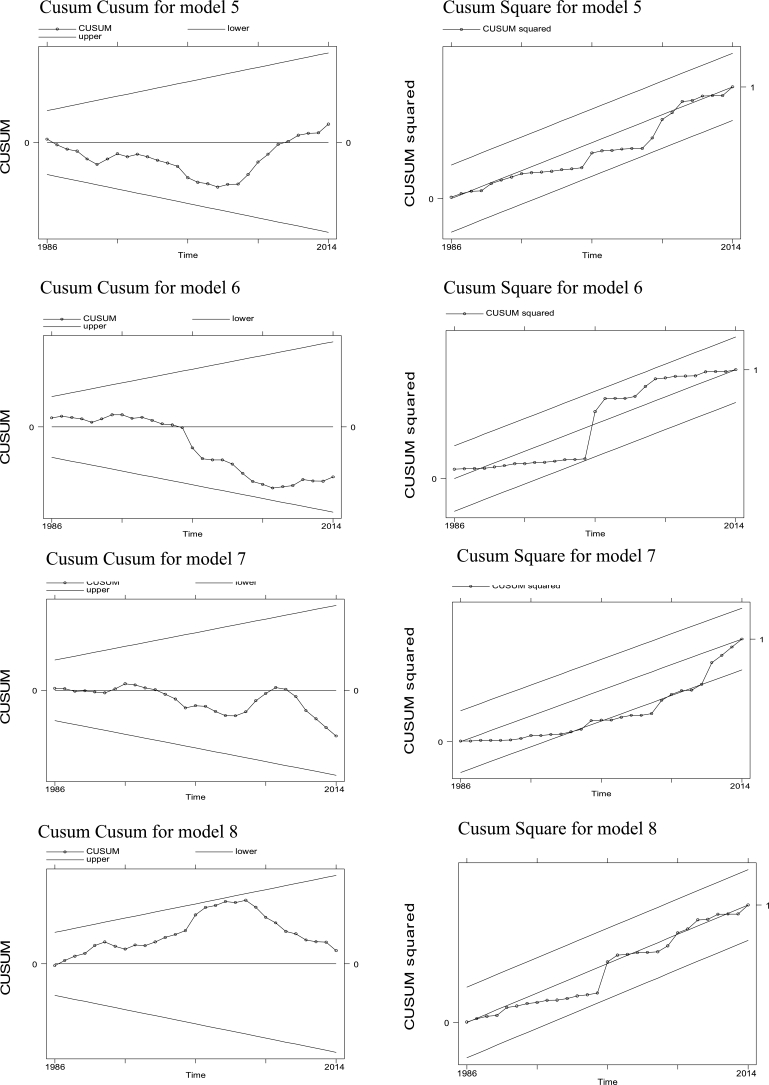
Cusum Cusum and Cusum Square graphs for models 5 to 8 of ARDL analysis. The top two pictures depict the Cusum cusum and Cusum square for model 5. Below them are the Cusum cusum and Cusum square for model 6, followed by Cusum cusum and Cusum squares of models 7 and 8. The post estimation results show that our coefficients are efficient as the graph lines of Cusum cusum and Cusum squares stay within the boundaries. The x-axis in all the graphs show time and y-axis of left-hand graphs show Cusum and the y-axis of right-hand graphs show Cusum squares.

To make sure that our coefficients are stable, we ran Cusum cusum and Cusum square post estimation tests. The results for all the models are shown in the Fig. 1 which depict that our coefficients are stable, and our results are robust.

Finally, Table 6 presents the Granger causality results for short term relationship between the variables under study. None of the series have bidirectional cause and effect relationship. As per the results, exports granger cause carbon emission and capital formation impacts carbon emission. No causal relationship has been found between carbon emission & energy use and exports and gross capital formation. Furthermore, both exports and capital formation granger cause energy use.

**Table 6:** Granger causality relationship.

Pair	Relationship
CO2 Emission and Exports	Exports to CO2 emission
CO2 Emission and GCF	GCF to CO2 emission
CO2 Emission and Energy use	No causal relationship
Exports and GCF	No causal relationship
Exports and Energy use	Exports to Energy use
GCF and Energy use	GCF to Energy use

## 5 Discussion under the policy perspectives

This section discusses the key findings of the outcomes related to our literature and then provides policy recommendations. This study has tried to find the relationship between exports, gross capital formation, energy use, and CO_2_ emission using the ARDL method from 1981-2020.

As provided above that the coefficient for the short-run proves that the exports adversely impact CO_2_ emission, which means that export is linked with lower CO_2_ emissions in Pakistan. In addition, these outcomes are in line with the study of Mao and He [26] for China who found that export of China promote contributed to the pollution reduction in 261 cities. The gross capital formation insignificantly impact CO_2_ emissions while the higher energy consumption causes higher CO_2_ emissions, which are consistent with the studies of Wu et al. [49] and Rokhmawati [36]. Gross capital formation as the dependent variable shows a negative association under the energy consumption, which means that the investment in renewable energy can be superior over long-run. This is in line with the study of Lin and Raza [24] who claimed that capital investment in the agriculture sector of Pakistan can significantly impact country's CO_2_ emissions reduction. Thus, in relation to the long run CO_2_ emissions and energy consumption relationship, the export plays an import role in Pakistan.

From the policy perspectives, Pakistan has adopted many policies targeting energy productivity in numerous productive sectors, such as industrial, transport and agriculture under Pakistan Economic Corridor (CPEC) and Nationally Determined Contribution (INDC) submitted on 12-November 2015. Also, the government will set an Action Plan for applying climate change mitigation estimations in various sector including energy sectors underlying gas imports, energy-saving awareness, sectorial energy consumption plan, and CO_2_ emissions reduction standard. This will rely on affordability, international investments, technical development, and capacity building. Moreover, in response to the reduction in carbon emissions from the energy and power sectors, application of new technologies and Dynamic Thermal Rating (DTR) system is necessary. For example, Teh et al. [44] explained the potential of applying the DTR system to increase the consistency of power systems under electricity networks (i.e., wind and smart gird technologies); Lai and Teh [19], [20] analyzed DTR system concentrated on transmission lines and networks and found that this system can provide higher ratings than the traditional ratings from 80-90% times; [19] examined the network topology optimization using DTR and battery storage systems for better wind productivity and its consistency. They found that the battery energy and energy ratings need to maintain the main security of supply standards in a large scale; Teh and Cotton [43] investigated the time-series data and found that DTR system is able to raise network reliability and permits for higher wind energy penetration; Teh et al. [44] analyzed the uncertainty impacts on the line failure model parameters using the DTR system reliability and found that compared model considers only the end of life failure impact of the transmission line, and Mashlakov et al. [27] analyzed the multi-service market optimization of battery energy storage system and found that there is uncertainty coming from coupled resources and processes.

Moreover, Mohamad et al. [29] analyzed the optimum allocation of battery energy storage systems for solar energy to reduce the CO_2_ emissions reduction. As solar energy is the imperative engine in lessening pollution in the world, thus, the power system has its ability to store solar power, which can be used later. This will avoid wastage and raise the utility profit. Presently, Pakistan intends to reduce up to 20% of its projected 2030 GHGs. Therefore, Pakistan should increase its energy efficiency and conservation using the solar reserves (6-7 hours sunlight), which can lessen the huge imported energy from different countries. Hence, significant progress in the energy substitutability process, the future renewable energy framework can be developed for country.

## 6 Conclusion and policy recommendations

The current research tried to give an empirical discussion about the association between CO_2_ emission, export of goods and services, gross capital formation and energy consumption. For this purpose, we used data from Pakistan for the period of 1981-2020 and employed the auto regressive distributive lag (ARDL) model to estimate the short-run and long-run relationship between above mentioned variables and causal relationship was determined by using Granger causality test. The results based on empirical findings show that:

First, in respect of time-series analysis of data, the Augmented Dickey Fuller, Philips and Perron and KSS tests were applied for the unit root test. The unit root tests results have proven that all the sequences in the present study have a unit root and are integrated at I (1) excluding gross capital formation, which is stationary at level.

Second, the empirical findings based on ARDL model present that carbon emissions reduce with increase in exports in case of Pakistan. These results are true both in short term as well as long term and CO_2_ emission returns to equilibrium at the speed of 54.9% in the long run. Also, the rise in CO_2_ emission is associated with lower export of goods and services. However, the causal association just moves from export of goods and services to CO_2_ emission. It is estimated that current period gross capital formation does not impact CO_2_ emission, however, lagged gross capital formation values significantly positively impact current exports. Generally, the findings from the current research give useful insights to those policy-makers so that they can better make their export policies to not only respond climate change but also encourage economic growth and raise employment opportunities.

Third, current values of energy consumption have positive impact on CO_2_ emission and lagged values have negative impact on the dependent variable in the short run. However, energy consumption only positively impacts carbon emission. The findings show that pollution would rise if the energy consumption would rise, and it will lower with increase in exports. This means that Pakistan can raise its exports to enhance the balance of payment because rising exports are beneficial for the country as well as have a minimal impact in harming the country's environment. Thus, based on empirical findings, current study suggests few policies for the progress of Pakistan. It is obvious that Pakistan's economy has been encouraged by maximum use of energy over the period, however, many factors that can increase or control over the pollution. Thus, to attain sustainable growth and a clean environment, the government and authorities should take serious steps in lessening CO_2_ emission. The implication of fossil fuel substitution with renewable energy resources is to be encouraged at elevated level, including their less cost associated with technologies. Present research obviously tells us that by growing the export, the CO_2_ emissions have less impact on environment; therefore, the government should focus on imported technologies, which really leads to the growth of Pakistan economy. Thus, Pakistan should export more like neighboring countries such as China and India. Conclusively, the related policies should have a clear alternative clean energy consumption target so as to attain energy productivity, which will automatically add to sustainable economic growth over the long run. This will not only impact economy but also help in reducing CO_2_ emissions. The findings from the current research give helpful insights to that policymaker so that they can better arrange their export policies to not only respond to environmental change but also enhance economic growth and rise employment opportunities.

In the end, the Dynamic Thermal Rating (DTR) developed transformation for the high renewable integration under the solar and wind is necessary, which will position power transformers close to the thermal limits. This will benefit due to low electricity price of renewables and raises the penetration of green energy into electricity markets [7].

Furthermore, this study is not without limitations: (1) for future economic development, the proportion of energy consumption in the overall demand will rise. To reduce energy share and increase export, modern technologies are required, which further needs to be discussed. This will impact on the supply side, which needs updates of each technical parameter. (2) Unfortunately, because of the unavailability of technical information, we turned our model towards economic, energy and climate change model, without seeing technical progress. To assess these key issues, further studies should be done to improve model assumptions employing new data and related information.

## Declarations

### Author contribution statement

Muhammad Umar: Conceived and designed the experiments; Performed the experiments; Analyzed and interpreted the data; Wrote the paper. Muhammad Yousaf Raza: Conceived and designed the experiments; Contributed reagents, materials, analysis tools or data; Wrote the paper. Yan Xu: Contributed reagents, materials, analysis tools or data.

### Funding statement

Yan Xu was supported by 10.13039/501100001809National Natural Science Foundation of China [12026239, 71872034], Scientific Research Project of Liaoning Education Department [LN2020J35], Research Project of Humanities and Social Sciences of Ministry of Education Planning Fund [22YJA910004].

### Data availability statement

Datasets related to this article can be found at https://databank.worldbank.org/reports.aspx?source=world-development-indicators, an open-source online data repository hosted at World Bank database.

### Declaration of interests statement

The authors declare no conflict of interest.

### Additional information

No additional information is available for this paper.
